# Seattle Protocol Is More Effective in Detection of Dysplasia Compared to Technology-Assisted Targeted Biopsies in Patients with Barrett’s Esophagus

**DOI:** 10.3390/jcm12072544

**Published:** 2023-03-28

**Authors:** Noam Peleg, Jacob E. Ollech, Steven Shamah, Boris Sapoznikov

**Affiliations:** 1 The Division of Gastroenterology, Rabin Medical Center, Petah-Tikva 49100, Israel; 2 Sackler Faculty of Medicine, Tel Aviv University, Tel Aviv 69978, Israel

**Keywords:** Barrett’s esophagus, surveillance, dysplasia, esophageal adenocarcinoma, advanced imaging

## Abstract

Background and aims: With the development of narrow-band imaging (NBI) in the endoscopic evaluation of patients with Barrett’s esophagus (BE), the role of random biopsies according to the Seattle protocol (SP) has been questioned. We aim to compare the utility of advanced imaging to SP in patients with BE. Methods: A prospective cohort of patients with proven BE was retrospectively analyzed. All biopsies were reviewed by an expert GI pathologist. Advanced imaging was tandemly used with SP in each endoscopic procedure. Results: A total of 155 out of 340 patients (45.5%) with BE were diagnosed with dysplasia during a median follow-up of 4.7 years (IQR 3.4–6.1 years) and were part of the statistical analysis. A total of 82 patients had a diagnosis of dysplasia at presentation, whereas 84 patients developed dysplasia during follow up. A total of 67 out of 82 patients with dysplasia at presentation (81.7%), and 65 out of 84 patients that were diagnosed with dysplasia during follow-up (77.4%) were diagnosed using SP. In addition, whereas all the events of EAC were diagnosed using targeted biopsies, 57.1% of the events of HGD and 86.3% of LGD were diagnosed using SP. Conclusion: Our findings demonstrate the significance of SP in the detection of low- and high-grade dysplasia in patients with BE. SP should remain the mainstay of endoscopic surveillance in this population.

## 1. Introduction

Gastroesophageal reflux disease (GERD) occurs when stomach contents reflux into the esophagus, causing irritating symptoms and complications [1]. GERD prevalence ranges from 8.7% to 33% in the Middle East and 18.1% to 27.8% in North America [2,3], and Barrett’s esophagus (BE) is a major complication of GERD, affecting up to 15% of the GERD population and 1–2% of the general population [4,5].

Patients with BE are at increased risk of developing esophageal adenocarcinoma (EAC), which is the eighth most common cancer and represents the sixth most common cause of cancer-related mortality worldwide [6]. The annual risk of conversion of BE to EAC is 0.33%, depending on the length of Barrett’s mucosa and the presence or absence of dysplasia [7,8]. In a large prospective cohort of the Scandinavian population with BE, the relative risk of development of EAC among patients with BE and low-grade dysplasia (LGD) was 4.8 (95% confidence interval 2.6–8.8) compared with those who did not have LGD, and was increased to 21.1 (95% confidence interval 17.8–24.7) among BE patients with high-grade dysplasia (HGD) at baseline [9]. It is believed that persistent local inflammation in the lower esophagus due to gastroesophageal reflux causes genetic and epigenetic alterations in the squamous mucosa over time, which may lead to acquisition of low- and high-grade dysplasia and eventually EAC [10]. Endoscopic surveillance programs were designed in order to detect esophageal dysplasia in its premalignant phase, or—if EAC is deemed to develop—in order to diagnose it at an earlier stage [11].

As the prevalence of EAC is on the rise [12], endoscopic methods have been established to increase the diagnostic yield of surveillance endoscopy in the diagnosis of dysplasia in BE patients. According to international guidelines, surveillance interval times differ according to the length of Barrett’s mucosa and the presence of dysplasia, as these are the two major risk factors for histologic progression. For nondysplastic BE (NDBE), the interval ranges between 3 and 5 years: every 5 years for short-segment BE, defined as less than 3 cm of Barrett’s mucosa; and every 3 years for long-segment BE, defined as more than 3 cm of Barrett’s mucosa. When dysplasia is detected, the intervals shorten, and surveillance endoscopy should be performed every 6 months, depending on the grade of dysplasia. In each surveillance biopsies should be taken systematically. The combination of Seattle protocol and chromoendoscopy, in which the topical application of acetic acid or Lugol’s iodine on the esophageal mucosa helps to enhance the contour and endoscopic morphology of the mucosa, is the current standard of care in dysplasia detection in patients with BE. According to the Seattle protocol, four-quadrant biopsies are taken separately every 1–2 cm of the Barrett’s mucosa length [13,14]. Nevertheless, the Seattle protocol is cumbersome [15,16], costly, and not immune to sampling error [17], primarily as less than 1% of the affected Barrett’s mucosa is evaluated in this method [18]. Accordingly, several advanced imaging modalities have been proposed to intensify the detection of dysplasia in BE, including narrow-band imaging (NBI) [19,20].

NBI uses the ability to limit the wavelength spectrum to visible blue light during endoscopy with specific optical filters. This in turn changes the depth of light penetration to the mucosa and optimizes mucosal imaging, enables the visualization of unique capillary patterns, and emphasizes angiogenesis as an early feature of dysplasia and neoplasia. However, the evaluation of NBI is mostly confined to selected high-risk populations in clinical trials, and real-world data on the yield of NBI in diagnosis of dysplasia, its usefulness in a real-world setting, and comparisons to the Seattle protocol in detection of dysplasia are currently scant and show conflicting results. Accordingly, current international guidelines do not recommend the use of advanced imaging modalities in routine surveillance endoscopies or to keep these techniques only as adjunctive methods, together with four-quadrant random biopsies [13,21].

This study aims to evaluate the utility of NBI and random four-quadrant biopsies according to the Seattle protocol in the first detection of dysplasia in patients with BE. 

## 2. Methods

In one BE referral center, a prospective cohort of patients with a histologic diagnosis of BE with dysplasia between January 2013 to December 2020 was analyzed retrospectively. Out of 340 patients diagnosed with BE during that period, 176 patients (51.76% of the total cohort) were not part of the analysis for not developing dysplasia during follow-up. The study was approved by the local institutional review board (RMC-170842, 1 February 2018).

### 2.1. Patient’s Assessment and Endoscopic Evaluation

Diagnosis of BE was defined using histologic criteria, and any dysplasia was confirmed in dedicated gastropathology joint meetings using established criteria for EAC, HGD, LGD, and NDBE. The date of diagnosis of BE was defined as the date of the index endoscopy, at which the histologic specimen with intestinal metaplasia with or without dysplasia was retrieved.

Endoscopic evaluation was performed using high-definition white light endoscopy (HD-WLE) in all cases and in all endoscopy procedures, including index and surveillance endoscopy procedures. The length of BE segment was recorded using the Prague classification during each endoscopy in all patients. Patients with BE segment of less than 3 cm were considered to have “short segment BE” and patients with BE mucosa above this threshold were considered to have “long segment BE”. Patients with Barrett’s mucosa less than 1 cm length were excluded according to international guidelines. Endoscopic follow-up was standardized in all patients according to international guidelines [14], and endoscopy was performed every 3–5 years in patients with NDBE. As endoscopic eradication therapy is reimbursed only in patients with three consecutive events of LGD, patients with LGD were followed every 6 months. In addition, patients with BE length of more than 10 cm were followed every 6 months. In case of diagnosis of “indefinite for dysplasia”, surveillance endoscopy was performed in an interval of 6 months. In case of a diagnosis of HGD or intramucosal carcinoma, the patient was referred to endoscopic eradication therapy of the Barrett’s mucosa. In case of detection of visible nodules of lesion during follow-up, the patients were referred to endoscopic resection (ER).

NBI assessment and Seattle protocol were tandemly used in each endoscopic procedure: the endoscopist examined the Barrett’s mucosa under HD-WLE followed by a full inspection of the mucosa with NBI. In case mucosal or vascular abnormalities were identified in HD-WLE or NBI, targeted ER or biopsies of the lesion were performed. In addition, mucosal biopsies were taken according to Seattle protocol in all cases and in all endoscopic procedures: four-quadrant biopsy specimens were taken at intervals of every 1 to 2 cm throughout the Barrett’s mucosa. All endoscopies were performed by BE specialist endoscopist [22] using the Olympus endoscopy system. In all the endoscopic procedures, we used GIF-H190 or GIF-H185 (Olympus, USA), although the option of near focus was not available. During follow-up, all patients were treated twice-daily with proton pump inhibitors (PPI) for reflux control. The specific type of PPI used was left to the discretion of the treating physician.

Dysplasia was classified as prevalent in the event of BE with dysplasia diagnosed during index endoscopy. However, if dysplasia was diagnosed during surveillance endoscopy in patients with previous NDBE or in case a patient with LGD progressed to HGD or EAC, it was considered to be an incident dysplasia.

### 2.2. Statistical Analysis

Statistical analysis was performed using SPSS version 25 (SPSS Inc., Chicago, IL, USA). Categorical and continuous variables were examined using the Student’s t-test, chi-square, or Fisher exact tests, as appropriate. A 2-sided *p*-value of less than 0.05 was considered statistically significant. The proportions of each category of dysplasia detected by the Seattle protocol and targeted biopsies using HD-WL and NBI were calculated. Next, we calculated the proportions of cases detected by Seattle protocol and targeted biopsies in NBI among cases where dysplasia was incident or prevalent and further stratified the results by dysplasia level (LGD, HGD, and EAC).

## 3. Results

**Figure 1:**
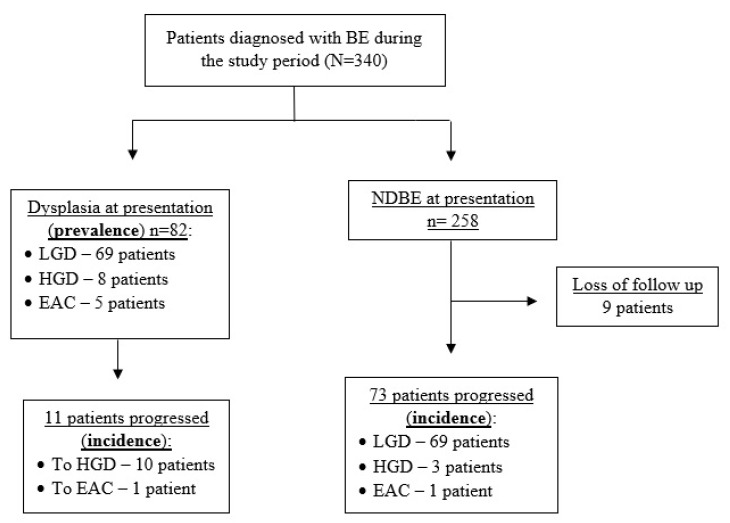
Study flow chart. NDBE—nondysplastic Barrett’s esophagus.

During the study period, 340 patients were diagnosed with BE and 1159 upper endoscopies were performed. A total of 155 patients were diagnosed with dysplasia during the study and were part of the statistical analysis (Figure 1). The median age at diagnosis of BE was 64.0 years (IQR 58-69), and most patients were males (123, 79.87%). Almost 50% of the study cohort had a hiatal hernia at index endoscopy, and the median hernia size was 3 cm. Median length of the BE segment at presentation was 2.0 cm (1.75–5), and 55 patients (35.48%) had a long segment of BE at presentation (Table 1).

BMI—body mass index, GERD—gastroesophageal reflux disease, BE—Barrett’s esophagus, PPI—proton pump inhibitor, EAC—esophageal adenocarcinoma, NDBE—nondysplastic Barrett’s esophagus, LGD—low-grade dysplasia, HGD—high-grade dysplasia.

A total of 82 patients (52.90%) were diagnosed with dysplastic BE or EAC at index endoscopy; 69 of them (84.14%) were diagnosed with LGD, 8 patients had HGD, and 5 had EAC at baseline (9.75% and 6.09%, respectively). Sixty-seven dysplasia events at baseline (81.71% of the cases) were invisible in HD-WL and NBI and were diagnosed using random biopsies with the Seattle protocol. Out of 15 prevalent dysplasia cases that were diagnosed using targeted biopsies, 10 (66.66%) were visible only in NBI and 5 (33.33%) with both HD-WL and NBI. Out of five prevalent cases of EAC, three were diagnosed with HD-WL, whereas in two cases the pathology was not seen in HD-WL endoscopy and was visible in NBI. In addition, out of eight prevalent cases of HGD, four cases (50%) were not visible, and were diagnosed using Seattle protocol (Table 2).

During median follow-up of 4.7 years (IQR 3.4–6.1 years), 84 cases of dysplasia were detected in surveillance endoscopies; 69 of them (82.14%) were diagnosed with LGD, 13 had HGD (15.47%), and 2 patients (2.38%) were diagnosed with EAC. The two incident cases of EAC were biopsied in targeted fashion after being diagnosed with NBI. However, out of 13 incidence cases of HGD, 8 cases (61.53%) were not visible and were diagnosed using the Seattle protocol, and out of 69 incident cases of LGD, 57 (82.61%) were diagnosed with random biopsy following the Seattle protocol (Table 3).

Three patients in our cohort were diagnosed as indefinite for dysplasia at index endoscopy, and the diagnosis was made using Seattle protocol biopsies in all three cases. Surveillance endoscopy was repeated at 6-month intervals as appropriate. During follow-up, all three of these patients progressed to LGD. The diagnosis of LGD was performed in random biopsies according to the Seattle protocol in all three patients.

Finally, after adding up, the Seattle protocol was the method of dysplasia diagnosis in 79.51% of cases of dysplasia diagnosed throughout the study. The utility of the Seattle protocol was most prominent in the events of LGD, in which 120 out of 139 cases of LGD were diagnosed with the Seattle protocol and were not seen in HD-WL or NBI. In addition, out of 34 cases of dysplasia that were detected in targeted biopsies, 13 cases (38.23%) were diagnosed with HD-WLE and 21 cases (61.76%) were detected solely by NBI. In addition, out of seven cases of prevalent and incident EAC, all were readily visible during endoscopy, although two of these cases were seen only using NBI.

## 4. Discussion

BE-associated dysplasia can be subtle, and sometimes barely visible. Hence, the quality of the endoscopic procedure and adequate sampling during surveillance are essential to optimize dysplasia detection and initiate the right therapeutic intervention. With the emergence of new and exciting advancements in endoscopy and new imaging modalities, some have argued that the Seattle protocol might be replaced as the sole standard of care in dysplasia surveillance of patients with BE [20]. If indeed feasible, this possibility can potentially reduce endoscopy time and pathology workload [23]. Our study, however, demonstrated that routine random four-quadrant biopsies according to the Seattle protocol is much more effective in the detection of dysplasia than targeted biopsies with HD-WL endoscopy and NBI. Almost 80% of all cases of detected dysplasia were diagnosed using random biopsies, with lower levels of dysplasia almost exclusively detected in this fashion.

As of 2016, a simple NBI classification system for identifying dysplasia from nondysplasia was constructed by the Barrett’s International NBI Group (BING) and demonstrated high diagnostic sensitivity and specificity, with overall accuracy of 85.4%, sensitivity of 80.4%, and specificity of 88.4% [24]. Non-experts, however, may not be able to achieve a similar diagnostic performance, since this validation was performed only by experts [25]. In addition, most studies evaluating the effectiveness of NBI have focused on very selected high-risk BE populations in which different degrees of dysplasia have been previously diagnosed. In our cohort, however, 176 patients did not develop dysplasia of any type during follow-up, and out of the included patients, which had prevalent or incident dysplasia, almost half of the cases had NDBE at presentation. We believe that these properties of our cohort add to the generalizability of our findings.

In our cohort, all cases of EAC were diagnosed using targeted biopsies, whereas lower levels of neoplasia and dysplasia were less readily visible and diagnosed primarily using random biopsies. This finding is in line with previous reports, which showed high variability in the sensitivity of NBI for detection of LGD, ranging from 10%∓87%, with a pooled PPV of 45% [26,27], although higher sensitivity and specificity were observed for the detection of HGD [28]. As LGD is a main risk factor for the development of EAC [29], and an indication for endoscopic eradication therapy in selected cases [13], we believe our observation strongly emphasizes the importance of the Seattle protocol as a gold standard in BE surveillance together with NBI or other chromoendoscopy.

Through a median follow-up of almost five years, we have demonstrated a detection rate of HGD and EAC of 0.96% per patient’s years of follow-up. As our population consisted of patients with different baseline dysplasia status, our results are comparable with previously published large population studies [19,30]. Surprisingly, more than half of the lesions with HGD in our cohort (13 out of 21 cases) were diagnosed with the use of random biopsies and were not seen with standard or advanced imaging. We believe that this finding further stresses the use of the Seattle protocol, even for the detection of advanced dysplasia. Furthermore, almost 40% of dysplasia events that were detected by targeted biopsies in our cohort were diagnosed using standard HD-WLE. It is therefore reasonable to suggest that advanced imaging with NBI plays only a modest role and should be kept for selected subtle lesions. Indeed, this conclusion reflects the concurrent international guidelines, which do not recommend the sole use of advanced imaging modalities routinely [13,21].

Although our finding supports the use of the Seattle protocol, NBI still remains valuable in the setting of BE surveillance, as it is readily available and may serve as an adjunct method for dysplasia detection. In addition, prior studies have shown that NBI has higher accuracy and sensitivity in dysplasia detection, with fewer biopsies taken and shorter procedural time compared to the Seattle protocol [15,16,31]. Additionally, previous studies have shown the cost effectiveness of using NBI in BE surveillance. In these studies, the expenditure on upgrading hospital and healthcare facilities with NBI technology can be balanced out by a significant reduction in costs from histological examination and cost saving from avoiding adverse events that might be related to oversampling [32]. Nevertheless, as more than half of the lesions with HGD in our cohort were missed by advanced imaging, we believe that additional technological advancements and studies are required before we can fully realize the clinical and economic benefits associated with NBI technology. An ongoing randomized control trial, examining high-resolution virtual chromoendoscopy versus the Seattle protocol (CONVERSE study) [33] is due in the near future and might shed light on this debate.

Several additional advanced technologies have been developed in an effort to improve dysplasia and neoplasia detection in patients with BE. For example, confocal laser endomicroscopy illuminates the esophageal mucosa after fluorescein injection while allowing real-time, high-definition imaging during surveillance endoscopy with proven high sensitivity and specificity [34]. Blue light imaging (BLI) is another endoscopic tool for visualization and delineation of BE-related neoplasia, and it may have additional value in BE surveillance [35]. Finally, artificial intelligence (AI) can be used for dysplasia and early carcinoma detection in BE, and several studies have validated a deep learning AI detection system that can detect and delineate early neoplasia [36]. These new technologies are rapidly advancing and have the potential to impact our approach on BE surveillance in the near future.

Our study is based on a large, prospectively managed cohort of BE patients, with all biopsies reviewed by an expert gastrointestinal pathologist with a relatively long follow-up period. Nonetheless, there are some limitations that warrant consideration. First, our data were retrospectively analyzed, and although the tandem evaluation of HD-WL and NBI is one advantage in our study, it might serve as potential confounding factor, as tandem use means that the Barrett’s mucosa is effectively inspected for longer time, which may increase dysplasia detection rates. Nevertheless, the dysplasia detection rates observed in our cohort are similar to previously published studies. Second, our study used standard nonmagnifying NBI, which may result in low dysplasia detection. However, magnifying NBI is not commonly found in general practice and is usually used only in selected centers. Accordingly, our results reflect the general practice in the field. Finally, the management of reflux symptoms was not the same for all study subjects, and the type of PPI used was not standardized during the study. In addition, we did not record the data regarding spatial distribution of dysplasia among study participants, and a total of nine patients had loss of follow-up during our study period. However, all patients were treated with PPIs twice daily, making sure reflux was treated; all biopsies taken were systematically reviewed by a dedicated gastrointestinal pathologist; and any dysplasia was reconfirmed in dedicated gastro-pathology joint meetings. As diagnostic characteristics such as sensitivity, specificity, and accuracy were not calculated in our analysis, further prospective studies are needed in order to fully assess the utility of NBI in the BE population and compare its dysplasia detection ability to the Seattle protocol.

In conclusion, we found that the Seattle protocol was the method of dysplasia detection in almost 80% of dysplasia cases in our cohort and in almost 40% of the cases of HGD. Our findings demonstrate the significance of the Seattle protocol in the detection of both low- and high-grade dysplasia compared to using HD-WL endoscopy and NBI in patients with BE. Consequently, the Seattle protocol should remain the mainstay of endoscopic surveillance in the general population of BE patients, together with dye-based or virtual chromoendoscopy.

## Figures and Tables

**Table 1:** Baseline characteristics of the study cohort; N = 155.

Clinical Parameters	
Age at diagnosis (years)	64.0 (58.0–69.0)
Gender—male	123 (79.30)
BMI	28.9 (26.9–31.8)
GERD at baseline	101 (65.16)
Family history of BE or EAC	6 (3.87)
Ever smoke	82 (52.90)
Alcohol use	9 (5.81)
PPI type	
Omeprazole	49 (31.61)
Lansoprazole	31 (20.00)
Esomeprazole	65 (41.93)
Lansoprazole	10 (6.45)
**Endoscopic and histologic parameters**	
BE length	
C (cm)	1.0 (0.0–2.0)
M (cm)	2.0 (1.0–3.0)
Long segment BE	55 (35.48)
Hiatal hernia	77 (49.67)
Hiatal hernia size (cm)	3.0 (3.0–4.0)
NDBE	70 (45.16)
Indefinite for dysplasia	3 (1.93)
LGD	69 (44.51)
HGD	8 (5.16)
EAC	5 (3.22)
Number of surveillance endoscopies during follow-up	3.0 (2.0–4.0)

The data for categorical variable include N (%); continuous variable includes median and 25–75% interquartile range.

**Table 2:** Prevalence of dysplasia (N = 82) stratified by biopsy type and dysplasia grade.

	Seattle Protocol	Targeted Biopsy			
			HD-WL	NBI	Total
LGD, N (%)	63 (91.3)		1 (16.6)	5 (83.3)	6 (8.7)
HGD, N (%)	4 (50.0)		3 (75.0)	1 (25.0)	4 (50.0)
EAC, N (%)	0 (0.0)		3 (60.0)	2 (40.0)	5 (100.0)
Total events, N (%)	67 (81.7)		7 (46.6)	8 (53.3)	15 (18.3)

LGD—low-grade dysplasia, HGD—high-grade dysplasia, EAC—esophageal adenocarcinoma, HD-WL—high-definition white light endoscopy, NBI—narrow-band imaging.

**Table 3:** Incidence of dysplasia (N = 84) stratified by biopsy type and dysplasia grade.

	Seattle Protocol	Targeted Biopsy			
			HD-WL	NBI	Total
LGD, N (%)	57 (82.6)		3 (25.0)	9 (75.0)	12 (17.4)
HGD, N (%)	8 (61.5)		3 (60.0)	2 (40.0)	5 (38.5)
EAC, N (%)	0 (0.0)		0 (0.0)	2 (100.0)	2 (100.0)
Total events, N (%)	65 (77.4)		6 (31.6)	13 (68.4)	19 (22.6)

LGD—low-grade dysplasia, HGD—high-grade dysplasia, EAC—esophageal adenocarcinoma, HD-WL—high-definition white light endoscopy, NBI—narrow-band imaging.

## Data Availability

Deidentified individual data, analytic methods, and study materials will be made available to other researchers in response to specific requests to the corresponding author and after local IRB authorization.
